# Comparative Analysis of Cardiac SPECT Myocardial Perfusion Imaging: Full-Ring Solid-State Detectors Versus Dedicated Cardiac Fixed-Angle Gamma Camera

**DOI:** 10.3390/medicina61040665

**Published:** 2025-04-04

**Authors:** Gytis Aleksa, Paulius Jaruševičius, Andrė Pacaitytė, Donatas Vajauskas

**Affiliations:** 1 Radiology Clinic, Medical Academy, Faculty of Medicine, Lithuanian University of Health Sciences, 44307 Kaunas, Lithuania; paulius.jarusevicius@kaunoklinikos.lt (P.J.); donatas.vajauskas@lsmu.lt (D.V.); 2 Department of Nuclear Medicine, Radiology Clinic, Lithuanian University of Health Sciences, Kaunas Clinics, 44307 Kaunas, Lithuania; 3 Faculty of Medicine, Lithuanian University of Health Sciences, 44307 Kaunas, Lithuania; andre.pacaityte@stud.lsmu.lt

**Keywords:** cardiac SPECT MPI, full-ring solid-state detectors, dedicated cardiac fixed-angle gamma camera

## Abstract

*Background and Objectives:* Single-photon emission computed tomography (SPECT) myocardial perfusion imaging (MPI) is a well-established technique for evaluating myocardial perfusion and function in patients with suspected or known coronary artery disease. While conventional dual-detector SPECT scanners have limitations in spatial resolution and photon detection sensitivity, recent advancements, including full-ring solid-state cadmium zinc telluride (CZT) detectors, offer enhanced image quality and improved diagnostic accuracy. This study aimed to compare the performance of Veriton-CT, a full-ring CZT SPECT system, with GE Discovery 530c, a dedicated cardiac fixed-angle gamma camera, in myocardial perfusion imaging and their correlation with coronary angiography findings. *Materials and Methods:* This was a prospective study that analyzed 21 patients who underwent MPI at the Department of Nuclear Medicine, Lithuanian University of Health Sciences, Kauno Klinikos. A one-day stress–rest protocol using 99mTc-Sestamibi was employed, with stress testing performed via bicycle ergometry or pharmacological induction. MPI was first conducted using GE Discovery 530c (GE Health Care, Boston, MA, USA), followed by imaging on Veriton-CT, which included low-dose CT for attenuation correction. The summed stress score (SSS), summed rest score (SRS), and summed difference score (SDS) were analyzed and compared between both imaging modalities. Coronary angiography results were retrospectively collected, and lesion-based analysis was performed to assess the correlation between imaging results and the presence of significant coronary artery stenosis (≥35% and ≥70% narrowing). Image quality and the certainty of distinguishing the inferior myocardial wall from extracardiac structures were also evaluated by two independent researchers with differing levels of experience. *Results*: Among the 14 patients included in the final analysis, Veriton-CT was more likely to classify MPI scans as normal (64.3%) compared to GE Discovery 530c (28.6%). Additionally, Veriton-CT provided a better assessment of the right coronary artery (RCA) basin, showing greater agreement with coronary angiography findings than GE Discovery 530c, although the difference was not statistically significant. No significant differences in lesion overlap were observed for the left anterior descending artery (LAD) or left circumflex artery (LCx) basins. Furthermore, the image quality assessment revealed slightly better delineation of extracardiac structures using Veriton-CT (Spectrum Dynamics Medical, Caesarea, Israel), particularly when evaluated by an experienced researcher. However, no significant difference was observed when assessed by a less experienced observer. *Conclusions:* Our findings suggest that Veriton-CT, with its full-ring CZT detector system, may offer advantages over fixed-angle gamma cameras in improving image quality and reducing attenuation artifacts in MPI. Although the difference in correlations with coronary angiography findings was not statistically significant, Veriton-CT showed a trend toward better agreement, particularly in the RCA basin. These results indicate that full-ring SPECT imaging could improve the diagnostic accuracy of non-invasive MPI, potentially reducing the need for unnecessary invasive angiography. Further studies with larger patient cohorts are required to confirm these findings and evaluate the clinical impact of full-ring SPECT technology in myocardial perfusion imaging.

## 1. Introduction

Single-photon emission computed tomography (SPECT) myocardial perfusion imaging (MPI) is widely used for diagnosing patients with known or suspected coronary artery disease, providing critical insights into both myocardial perfusion and function [1]. According to European Society of Cardiology (ESC) guidelines, SPECT imaging should be performed for patients with suspected chronic coronary syndrome (CCS) with a moderate or high pre-test likelihood of obstructive coronary artery disease CAD (15–85%) or known CCS [2]. During an MPI scan, a gamma camera detects the distribution of an intravenously administered radiotracer to assess coronary blood flow within the myocardium. Traditional MPI techniques involve rotating a gamma camera along a 180-degree arc to capture multiple planar projections [3]. Due to their physical and mechanical limitations, conventional dual-detector SPECT scanners exhibit relatively low spatial resolutions and photon-detection sensitivity. However, recent advancements have led to the development of solid-state direct-conversion detectors, particularly cadmium zinc telluride (CZT)-based SPECT systems, which provide improved quantitative imaging compared to traditional Anger-based cameras [4]. The ongoing evolution of nuclear medicine instrumentation has been driven by the need to enhance image quality, quantification accuracy, and clinical utility [5].

Across Europe, nuclear medicine facilities have increasingly adopted next-generation dedicated cardiac gamma cameras for myocardial perfusion studies. Among the most widely used systems are the D-SPECT (Spectrum Dynamics, Morges, Switzerland) and the Alcyone (General Electric Medical Systems, Milwaukee, WI, USA). Although these systems share CZT-based detectors, they differ in terms of detector configuration, collimation methods, and image reconstruction algorithms [6]. Compared to traditional Anger cameras, dedicated cardiac SPECT cameras using CZT detectors offer significant advantages, including higher spatial resolution, shorter scan times, and lower radiotracer doses, all while maintaining superior image quality. Notably, the reduction in scan time improves patient comfort and decreases the risk of motion artifacts, which are common in MPI interpretation [7].

Recently, new-generation ring-shaped SPECT systems—such as Veriton (Spectrum Dynamics) and StarGuide (GE Healthcare)—have been introduced. These designs resemble positron emission tomography (PET) systems, featuring multiple stationary CZT detectors arranged in a circular geometry. Veriton, for instance, incorporates 12 distinct CZT detectors, positioned within a fixed ring configuration. A key innovation of this system lies in the mobility of individual detector units, which reduce the distance to the targeted anatomical region, ultimately enhancing image resolution and diagnostic accuracy [8].

Studies evaluating the Veriton CZT-based system against conventional Anger cameras have demonstrated superior sensitivity, energy resolution, and image contrast [9]. However, MPI remains susceptible to artifacts. The most common challenges include patient motion artifacts, as well as extra-cardiac activity artifacts from sub-diaphragmatic radiotracer uptake in the liver and gastrointestinal tract, which can interfere with inferior wall assessment. Other well-documented artifacts include breast attenuation artifacts, particularly in women with large breasts, and diaphragmatic attenuation, which frequently affects obese male patients [10].

The use of full-ring solid-state CZT detectors has the potential to mitigate these artifacts and further improve image quality compared to dedicated cardiac fixed-angle gamma cameras. Therefore, the aim of our study was to compare MPI results obtained using the Veriton-CT system with those from a dedicated cardiac fixed-angle gamma camera in the same patient cohort.

## 2. Materials and Methods

This was a prospective, small-sample study in which data from 21 patients were analyzed. All patients were referred to the Department of Nuclear Medicine at the Hospital of Lithuanian University of Health Sciences Kauno Klinikos for myocardial perfusion imaging (MPI) and were randomly selected for this study. The patients selected for the study were indicated for myocardial perfusion scintigraphy under the direction of their cardiologist for suspected ischemic heart disease and a low pre-test probability of coronary artery disease. A one-day stress–rest protocol with 99mTc-Sestamibi was used, with an injected activity of 2.5 MBq/kg for stress imaging and 2.5 times that dose for rest imaging. The stress test was performed either by bicycle ergometry or via a pharmacological test with adenosine.

MPI was initially performed using the Discovery NM 530c gamma camera, 30 min after Sestamibi injection, followed by scanning with Veriton-CT, which included low-dose CT for attenuation correction. The summed stress score (SSS), summed rest score (SRS), and summed difference score (SDS) results were gathered from both imaging systems and compared.

MPI results were interpreted as follows:

- The SSS was classified as normal if <4, mildly abnormal if 4–8, moderately abnormal if 9–13, and significantly abnormal if >13.
- The SDS was classified as normal if <2, mildly abnormal if 2–4, moderately abnormal if 5–6, and significantly abnormal if >6.

Additionally, we retrospectively collected data on the most recent coronary angiography or coronary CT angiography performed on these patients. Seven patients were excluded from further data analysis due to the absence of coronary imaging results—neither computed tomography angiography (CTA) nor invasive coronary angiography (ICA) data were available.

From the coronary angiography, we gathered information about lesions in coronary basins. The angiography results were categorized as normal or abnormal based on the percentage of coronary artery narrowing. Abnormal results were further divided into two groups:

- Lesions with at least 35% narrowing.
- Lesions with at least 70% narrowing.

We performed a lesion-based analysis to compare the MPI results obtained from Veriton-CT with those from a dedicated cardiac fixed-angle gamma camera, assessing their agreement with coronary angiography findings.

**Figure 1:**
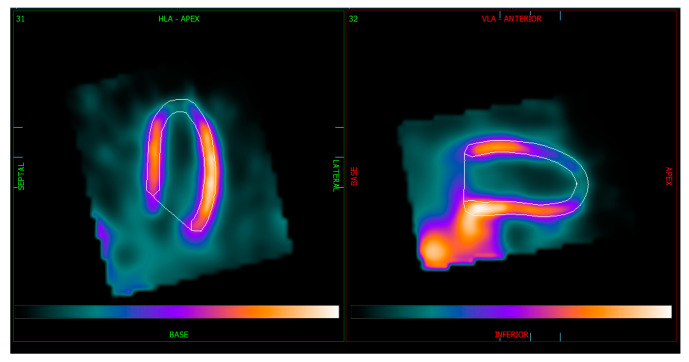
Veriton-CT SPECT images of LV myocardium in horizontal and vertical long axes with merged structures.

**Figure 2:**
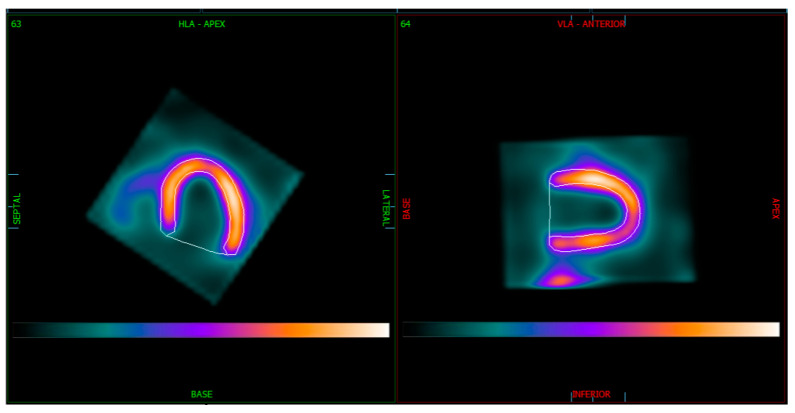
Veriton-CT SPECT images of LV myocardium in horizontal and vertical long axes with delineated contours.

We also evaluated image quality and the certainty of distinguishing the inferior myocardial wall from extracardiac structures when analyzing MPI from both Veriton-CT and the Discovery NM 530c gamma camera. To assess image quality, each MPI SPECT study was classified into one of two categories:

- Merged extracardiac structures with the left ventricular inferior wall (Figure 1).
- Clearly delineated extracardiac structures from the left ventricular inferior wall (Figure 2).

The image quality assessment was conducted by two researchers—one with extensive experience and the other with limited experience.

Statistical analysis was performed using IBM SPSS (version 29.0.1.0). The chi-square test was used if the expected cell count was less than 33.3%, while Fisher’s exact test was applied otherwise. A *p*-value of <0.05 was considered statistically significant.

This prospective study was approved by the Kaunas Regional Biomedical Research Ethics Committee (Project ID: BE 2-1, approval date: 17 February 2023).

## 3. Results

Of the fourteen patients included in the analysis, ten (71.4%) were male and four (28.6%) were female.

Comparing SSS results between the two imaging modalities showed that nine out of fourteen patients (64.3%) scanned using Veriton-CT were interpreted as having normal perfusion, whereas only four out of fourteen patients (28.6%) were classified as normal using the GE Discovery 530c. The GE Discovery 530c identified mildly abnormal perfusion in six patients (42.9%), while Veriton-CT categorized only three patients (21.4%) as mildly abnormal. Veriton-CT was more likely to classify scans as normal compared to the GE Discovery 530c (*p* = 0.210) (Table 1).

The comparison of SDS results revealed a similar trend. Veriton-CT classified normal perfusion in nine patients (64.3%), while GE Discovery 530c identified normal perfusion in only three patients (21.4%). GE Discovery 530c categorized six patients (42.9%) as mildly abnormal, whereas Veriton-CT classified only two patients (14.3%) as mildly abnormal. Veriton-CT was more likely to classify scans as normal compared to the GE Discovery 530c (*p* = 0.118) (Table 2).

The analysis of coronary artery lesions from coronary angiography showed varying prevalence across different arterial basins. Lesions with ≥35% narrowing were detected in 64.3% of cases in the right coronary artery (RCA) and left circumflex artery (LCx) and in 50.0% of cases in the left anterior descending artery (LAD). Lesions with ≥70% narrowing were observed in 28.6% of cases in the RCA and in 35.7% of cases in both the LAD and LCx (Table 3).

The overlap between coronary angiography findings (≥70% narrowing) and imaging results (SSS > 3) from Veriton-CT and GE Discovery 530c showed a slightly higher overlap for the RCA using Veriton-CT, though the difference was not statistically significant. For the LAD and LCx, overlap was similar between both imaging modalities (Table 4).

The overlap between coronary angiography findings (≥35% narrowing) and imaging results (SSS > 3) from Veriton-CT and GE Discovery 530c showed a slightly higher overlap for the RCA using GE Discovery 530c, though the difference was not statistically significant. For the LAD and LCx, the overlap was similar between both imaging modalities (Table 5).

The overlap between coronary angiography findings (≥70% narrowing) and imaging results (SDS > 1) from Veriton-CT and GE Discovery 530c showed a slightly higher overlap for the RCA using Veriton-CT, though the difference was not statistically significant. For the LAD and LCx, the overlap was similar between both imaging modalities (Table 6).

The overlap between coronary angiography findings (≥35% narrowing) and imaging results (SDS > 1) from Veriton-CT and GE Discovery 530c showed a slightly higher overlap for the RCA using Veriton-CT, though the difference was not statistically significant. GE Discovery 530c showed a slightly higher overlap for the LAD. For LCx, the overlap was similar between both imaging modalities (Table 7).

The evaluation of image quality and the relationship between the inferior myocardial wall and extracardiac structures showed slightly better results for Veriton-CT, particularly when assessed by the researcher with extensive experience. Merged extracardiac structures were identified in four out of fourteen cases using GE Discovery 530c and two out of fourteen cases using Veriton-CT. When assessed by the researcher with limited experience, both imaging modalities showed merged structures in two out of fourteen cases. The difference in image quality assessment between Veriton-CT and GE Discovery 530c was not statistically significant (*p* > 0.05) (Table 8).

## 4. Discussion

Dedicated cardiac-centered fixed-angle gamma cameras have demonstrated improved image quality, faster acquisition times, and superior diagnostic performance compared to conventional dual-head SPECT systems. However, these systems also present certain pitfalls and artifacts, particularly in obese patients, where diaphragmatic and soft-tissue attenuation, as well as extracardiac activity, may reduce test specificity in myocardial perfusion imaging (MPI) [11].

The presence of tissue attenuation degrades the sharpness and contrast-to-noise ratio of myocardial perfusion scintigraphy. Attenuation artifacts are also present in D-SPECT, another cardiac-centered gamma camera, which is known to be affected by diaphragm-induced attenuation that primarily impacts the apical segments of the inferior and inferolateral walls due to the semi-supine patient position during acquisition [10]. Additionally, rim-filter artifact scans result in artificially low signal intensities in the inferior wall, further complicating diagnostic accuracy [10].

Attenuation correction remains a significant challenge in routine clinical practice, as it can compromise image quality and introduce diagnostic uncertainty [12]. The full-ring SPECT system has been recognized for its enhanced sensitivity, reduced acquisition times, and improved resolution. The Veriton-CT system represents a significant advancement in conventional SPECT/CT imaging, offering sensitivity and resolution comparable to contemporary PET/CT systems [6].

One key advantage of 360-degree image acquisition is its ability to enhance accuracy and reduce image artifacts and geometric distortion compared to 180-degree acquisitions, ultimately leading to higher image quality and improved diagnostic value [13]. A study investigating the effect of the acquisition arc on artifact occurrence in myocardial perfusion SPECT concluded that 360-degree sampling significantly reduces attenuation artifacts compared to 180-degree acquisition [12].

The supine position is the most commonly used patient posture for MPI scans. However, it is associated with diaphragmatic attenuation of the left ventricular inferior wall (more prevalent in males) and breast attenuation of the left ventricular anterior wall (more common in females) [14]. Several strategies have been proposed to mitigate these attenuation artifacts, including prone positioning and hybrid SPECT/CT imaging. The acquisition of additional prone SPECT images, as well as upright imaging, has been suggested as a means to differentiate between attenuation artifacts and true hypoperfusion defects, thereby enhancing the overall diagnostic accuracy and specificity of MPI [15].

The acquisition of supine–prone images is practical and cost-effective, as it does not expose patients to additional radiation [14]. However, prone positioning may be uncomfortable for certain patient populations, including women and severely obese individuals [15]. In the absence of a CT attenuation correction component, misinterpretation of false perfusion defects may occur due to an artificial decrease in signal intensity caused by surrounding peri-myocardial soft-tissue structures such as the diaphragm, breasts, and chest wall [16].

The use of hybrid cardiovascular imaging has been increasing in recent years, as it enhances diagnostic accuracy, prognostic value, and risk stratification in patients with suspected coronary artery disease (CAD) [17]. One of the primary indications for low-dose CT in MPI is tissue attenuation correction. While attenuation correction CT is essential for PET MPI, its clinical integration into SPECT MPI protocols remains inconsistent across institutions. Nonetheless, attenuation correction has been shown to significantly improve diagnostic accuracy in SPECT MPI, particularly among obese patients [17].

Beyond the cardiovascular domain, hybrid imaging has been adopted across various medical disciplines, expanding the scope of complex disease imaging. It has proven effective for whole-body staging and re-staging in oncology, pulmonary embolism diagnosis, parathyroid imaging, and preoperative surgical planning [18,19,20]. However, hybrid imaging is associated with higher radiation exposure and should only be employed when there is a clear clinical benefit. Radiation exposure depends on multiple factors, including scanner type, patient geometry (e.g., chest diameter), scan range, and cardiac rhythm. While unnecessary radiation should be minimized, an additional radiation dose may be justified if hybrid imaging significantly enhances diagnostic accuracy and prognostic value [17].

Our study demonstrated that Veriton-CT, a full-ring SPECT gamma camera, provided a better assessment of the inferior myocardial wall compared to a dedicated cardiac fixed-angle gamma camera. This was particularly evident in the evaluation of right coronary artery (RCA) lesions, where Veriton-CT results were more consistent with coronary angiography than those from GE Discovery 530c. The differences in RCA basin overlap were not statistically significant, likely due to the small sample size. Lesion overlap with coronary angiography in other coronary artery basins (LAD and LCx) did not differ significantly between the two imaging modalities.

Another positive feature of novel CZT gamma cameras is that, with improved spatial resolution and inferior wall delineation, the images are easier to read, which boosts readers’ confidence, may reduce inter-reader variability, and lowers the learning curve for less experienced nuclear medicine physicians. In addition, the possibility of scan time and injected activity reduction can increase patient throughput and therefore reduce long waiting times for MPS. Furthermore, as these full-ring SPECT gamma cameras can be used for other purposes beyond cardiology, they could be a very cost-effective option for centers that perform fewer myocardial perfusion scintigraphies.

The clinical implications of improving non-invasive testing accuracy are substantial: by reducing false-positive perfusion abnormalities, more patients will be correctly diagnosed without requiring invasive angiography. This, in turn, may lead to fewer unnecessary angiography referrals, reduced radiation exposure, fewer unnecessary procedures, and decreased patient wait times for those who truly require invasive coronary assessment.

In this study, we explored the possible advantages of novel CZT cameras for MPS. For future research opportunities, it would be interesting to compare these results with other, more precise functional imaging techniques, such as myocardial perfusion PET or MRI. Previously, similar small-sample studies have been performed for the validation of cardiac-dedicated CZT cameras, compared to PET with various perfusion tracers. In addition, there is a rising need for an effective tool to diagnose non-obstructive CAD, which could previously only be carried out with PET or MRI imaging. Novel CZT technology enables us to perform dynamic MPS studies and evaluate myocardial blood flow and myocardial flow reserve; however, such studies on a full-ring SPECT system are still scarce.

## 5. Conclusions

Our study results demonstrated that Veriton-CT was more likely to classify MPI scans as normal compared to GE Discovery 530c, with a higher proportion of normal perfusion interpretations. Additionally, Veriton-CT provided a better assessment of the right coronary artery (RCA) basin, showing greater agreement with coronary angiography findings compared to GE Discovery 530c.

These findings suggest that Veriton-CT, with its full-ring CZT detector system, may offer advantages over fixed-angle gamma cameras in improving image quality and reducing attenuation artifacts in MPI. While the results indicate a trend toward improved correlations with coronary angiography, further studies with larger patient cohorts are necessary to confirm these findings and to evaluate the clinical impact of full-ring SPECT technology in routine myocardial perfusion imaging.

## 6. Limitations

Our study has several limitations that should be acknowledged:

The study included a relatively small number of patients (*n* = 14 for final analysis), which limits the statistical power to detect significant differences between the imaging modalities. A larger cohort is needed to confirm these findings and improve the reliability of the results.

The study was conducted in a single institution, which may limit the generalizability of the findings to other clinical settings with different imaging protocols, equipment, or patient populations.

The study focused on diagnostic agreement between SPECT MPI and coronary angiography but did not evaluate patient outcomes, such as the impact of imaging findings on clinical decision-making or long-term prognostic value.

Since only patients who underwent both imaging modalities and had available coronary angiography data were included, selection bias may have influenced the results. Patients with borderline or uncertain findings might have been excluded.

Although Veriton-CT showed a trend toward better agreement with coronary angiography, particularly in the RCA, the differences were not statistically significant (*p* > 0.05), which may be attributed to the small sample size.

While Veriton-CT included low-dose CT for attenuation correction, GE Discovery 530c did not, which may have influenced the results. However, the impact of this difference was not explicitly evaluated.

Image interpretation was performed by two researchers with different levels of experience. Although efforts were made to ensure consistency, intra- and interobserver variability could still impact the interpretation of results.

## Figures and Tables

**Table 1:** Comparison of SSS results between Veriton-CT and GE Discovery 530c.

SSS Classification	Veriton-CT	GE Discovery 530c
Normal perfusion	9 (64.3%)	4 (28.6%)
Mildly abnormal perfusion	3 (23.4%)	6 (42.9%)
Moderately abnormal perfusion	0 (0%)	1 (7.1%)
Significantly abnormal perfusion	2 (14.3%)	3 (21.4%)

SSS—summed stress score.

**Table 2:** Comparison of SDS results between Veriton-CT and GE Discovery 530c.

SDS Classification	Veriton-CT	GE Discovery 530c
Normal perfusion	9 (64.3%)	3 (21.4%)
Mildly abnormal perfusion	2 (14.3%)	6 (42.9%)
Moderately abnormal perfusion	2 (14.3%)	2 (14.3%)
Significantly abnormal perfusion	1 (7.1%)	3 (21.4%)

SDS—Summed Rest Score.

**Table 3:** Coronary angiography findings.

Coronary Artery	Lesion of ≥35% Narrowing	Lesion of ≥70% Narrowing
RCA	9 (64.3%)	4 (28.6%)
LAD	7 (50.0%)	5 (35.7%)
LCx	9 (64.3%)	5 (35.7%)

RCA—right coronary artery, LAD—left anterior descending artery, LCx—left circumflex artery.

**Table 4:** Overlap of coronary angiography lesions (≥70%) with Veriton-CT and GE Discovery 530c based on summed stress score.

Coronary Angiography ≥ 70% (SSS)	Veriton-CT, Overlap	GE Discovery 530c, Overlap	*p* Value
RCA	10 (71.4%)	8 (57.1%)	0.430 ^a^
LAD	9 (64.3%)	9 (64.3%)	1.000 ^a^
LCx	9 (64.3%)	8 (57.1%)	0.699 ^a^

^a^—Chi square test. RCA—right coronary artery, LAD—left anterior descending artery, LCx—left circumflex artery.

**Table 5:** Overlap of coronary angiography lesions (≥35%) with Veriton-CT and GE Discovery 530c based on summed stress score.

Coronary Angiography ≥ 35% (SSS)	Veriton-CT, Overlap	GE Discovery 530c, Overlap	*p* Value
RCA	5 (35.7%)	7 (50.0%)	0.445 ^a^
LAD	7 (50.0%)	7 (50.0%)	1.000 ^a^
LCx	5 (35.7%)	4 (28.6%)	1.000 ^b^

^a^—Chi square test; ^b^—Fisher’s exact test. RCA—right coronary artery, LAD—left anterior descending artery, LCx—left circumflex artery.

**Table 6:** Overlap of coronary angiography lesions (≥70%) with Veriton-CT and GE Discovery 530c based on summed difference score.

Coronary Angiography ≥ 70% (SDS)	Veriton-CT, Overlap	GE Discovery 530c, Overlap	*p* Value
RCA	8 (57.1%)	6 (42.9%)	0.450 ^a^
LAD	8 (57.1%)	9 (64.3%)	0.699 ^a^
LCx	8 (57.1%)	7 (50.0%)	0.705 ^a^

^a^—Chi square test. RCA—right coronary artery, LAD—left anterior descending artery, LCx—left circumflex artery.

**Table 7:** Overlap of coronary angiography lesions (≥35%) with Veriton-CT and GE Discovery 530c based on summed difference score.

Coronary Angiography ≥ 35% (SDS)	Veriton-CT, Overlap	GE Discovery 530c, Overlap	*p* Value
RCA	7 (50.0%)	5 (35.7%)	0.445 ^a^
LAD	6 (42.9%)	8 (57.1%)	0.450 ^a^
LCx	4 (28.6%)	5 (35.7%)	1.000 ^b^

^a^—Chi square test; ^b^—Fisher’s exact test. RCA—right coronary artery, LAD—left anterior descending artery, LCx—left circumflex artery.

**Table 8:** Comparison of image quality between Veriton-CT and GE Discovery 530c.

Image Quality	Imaging Modality	Researcher with Extensive Experience	Researcher with Limited Experience
Clearly delineated structures	Veriton-CT	12	12
	GE Discovery 530c	10	12
Merged structures	Veriton-CT	2	2
	GE Discovery 530c	4	2

## Data Availability

The original contributions presented in this study are included in the article. Further inquiries can be directed to the corresponding author.

## Abbreviations

The following abbreviations are used in this manuscript:

SPECT	Single-photon emission computed tomography
MPI	Myocardial perfusion imaging
CZT	Cadmium zinc telluride
SSS	Summed stress score
SRS	Summed rest score
SDS	Summed difference score
LAD	Left anterior descending artery
LCx	Left circumflex artery
RCA	Right coronary artery
CTA	Computed tomography angiography
ICA	Invasive coronary angiography
PET	Positron emission tomography
CAD	Coronary artery disease

## References

[1] Slomka P.J., Miller R.J.H., Hu L.H., Germano G., Berman D.S. (2019). Solid-State Detector SPECT Myocardial Perfusion Imaging. J. Nucl. Med..

[2] Vrints C.J., Andreotti F., Koskinas K.C., Rossello X., Adamo M., Ainslie J., Banning A.P., Budaj A., Buechel R.R., Chiariello G.A. (2024). 2024 ESC Guidelines for the management of chronic coronary syndromes. Eur. Heart J..

[3] Adnan G., Rahman M.N. (2024). Nuclear Medicine SPECT Scan Cardiovascular Assessment, Protocols, and Interpretation. StatPearls [Internet].

[4] Huh Y., Yang J., Dim O.U., Cui Y., Tao W., Huang Q., Gullberg G.T., Seo Y. (2021). Evaluation of a variable-aperture full-ring SPECT system using large-area pixelated CZT modules: A simulation study for brain SPECT applications. Med. Phys..

[5] van der Meulen N.P., Strobel K., Lima T.V.M. (2021). New Radionuclides and Technological Advances in SPECT and PET Scanners. Cancers.

[6] Wacholz C., Hruska C., O’Connor M. (2020). Veriton Multi-CZT Detector SPECT/CT System Acceptance Testing. J. Nucl. Med..

[7] Desmonts C., Bouthiba M.A., Enilorac B., Nganoa C., Agostini D., Aide N. (2020). Evaluation of a new multipurpose whole-body CzT-based camera: Comparison with a dual-head Anger camera and first clinical images. EJNMMI Phys..

[8] Morelle M., Bellevre D., Hossein-Foucher C., Manrique A., Bailliez A. (2020). First comparison of performances between the new whole-body cadmium-zinc-telluride SPECT-CT camera and a dedicated cardiac CZT camera for myocardial perfusion imaging: Analysis of phantom and patients. J. Nucl. Cardiol..

[9] Allie R., Hutton B.F., Prvulovich E., Bomanji J., Michopoulou S., Ben-Haim S. (2016). Pitfalls and artifacts using the D-SPECT dedicated cardiac camera. J. Nucl. Cardiol..

[10] Hyafil F., Gimelli A., Slart R.H., Georgoulias P., Rischpler C., Lubberink M., Sciagra R., Bucerius J., Agostini D., Verberne H.J. (2019). EANM procedural guidelines for myocardial perfusion scintigraphy using cardiac-centered gamma cameras. Eur. J. Hybrid Imaging.

[11] Gebhard C., Fuchs T.A., Ghadri J.R., Stehli J., Kazakauskaite E., Herzog B.A., Pazhenkottil A.P., Gaemperli O., Philipp A. (2012). Cadmium-Zinc-Telluride Myocardial Perfusion Imaging in Obese Patients Michael Fiechter. Kaufmann J. Nucl. Med..

[12] Dimitris A., Trifon S., Theodoros S., Costas G., Christos S., Pavlos V. (2008). Comparison between 180° and 360° acquisition arcs with and without correction by CT-based attenuation maps in normal hearts at rest. Nucl. Med. Commun..

[13] Freeman M.R., Konstantinou C., Barr A., Greyson N.D. (1998). Clinical comparison of 180-degree and 360-degree data collection of technetium 99m sestamibi SPECT for detection of coronary artery disease. J. Nucl. Cardiol..

[14] Ramos S.M.O., Glavam A.P., de Brito A.S.X., Kubo T.T.A., Tukamoto G., Sampaio D.D.C.P., de Sá L.V. (2020). Prone Myocardial Perfusion Imaging and Breast Attenuation: A Phantom Study. Curr. Med. Imaging Rev..

[15] Tawakol A.E., Tantawy H.M., Elashmawy R.E., Abdelhafez Y.G., Elsayed Y.M. (2021). Added Value of CT Attenuation Correction and Prone Positioning in Improving Breast and Subdiaphragmatic Attenuation in Myocardial Perfusion Imaging. J. Nucl. Med. Technol..

[16] Chopra S., Singh S.S., Sood A., Parmar M., Parihar A.S., Vadi S.K., Mittal B.R. (2023). Comparison of positional artifacts in myocardial perfusion imaging in supine and semi-reclining positions using dedicated D-SPECT cardiac camera: Validation using CT-based attenuation correction. J. Nucl. Cardiol..

[17] Caobelli F., Dweck M.R., Albano D., Gheysens O., Georgoulias P., Nekolla S., Lairez O., Leccisotti L., Lubberink M., Massalha S. (2025). Hybrid cardiovascular imaging. A clinical consensus statement of the European Association of Nuclear Medicine (EANM) and the European Association of Cardiovascular Imaging (EACVI) of the ESC. Eur. J. Nucl. Med. Mol. Imaging.

[18] Becker J., Schwarzenböck S.M., Krause B.J. (2020). FDG PET hybrid imaging. Recent Results Cancer Res..

[19] Simanek M., Koranda P. (2016). The benefit of personalized hybrid SPECT/CT pulmonary imaging. Am. J. Nucl. Med. Mol. Imaging.

[20] Cytawa W., Teodorczyk J., Lass P. (2013). Advantages of hybrid SPECT-CT imaging in preoperative localization of parathyroid glands in a patient with secondary hyperparathyroidism: A case report. Pol. J. Radiol..

